# Supplementary material for: Mapping and characterising electronic palliative care coordination systems and their intended impact: A national survey of end-of-life care commissioners
Source: PLoS One. 2022 Oct 14;17(10):e0275991. doi: 10.1371/journal.pone.0275991 (PMC9565729; doi:10.1371/journal.pone.0275991)
Supplement: S1 File — (PDF) [file pone.0275991.s002.pdf]

# Electronic Palliative Care Co-ordination Systems (EPaCCS):
## Evaluating their implementation & optimising future service provision

Survey of CCG Commissioners of Palliative Care and End of Life services in England

Survey open December 2020 until April 2021

Three versions of the Survey:

CCGs with an operational EPaCCS ........ 2
CCGs at the planning stage of implementing an EPaCCS ........ 16
CCGs without an EPaCCS ........ 24

# CCGs with an operational EPaCCS

## Electronic Palliative Care Co-ordination Systems (EPaCCS): Evaluating their implementation & optimising future service provision

This survey aims to capture progress towards implementing EPaCCS across England. It will help to understand how they are being used and identify how local implementation can be best supported. It will also support future development of the national information standard and national guidance.

This research is being undertaken by researchers at the University of Leeds, Kings College London and the Hull York Medical School. We are working in partnership with NHS England and Improvement and Public Health England.

**The survey comprises of two parts:**

1. Questions about implementation and use of EPaCCS - this takes around **10-15** minutes and should be completed by a CCG end of life care lead or a nominated representative.
2. Questions about numbers of patients registered - this takes around **20-30 minutes** and you can nominate a data manager to complete this part.

If you have any questions, please contact:


Chief Investigator

Research Fellow


**Your participation will enable us to provide you with a report of benchmarking data describing models of implementation and reported levels of uptake, and comparing local data with anonymised data from other CCGs that complete the survey.**


This study has been approved by University of Leeds School of Medicine Ethics committee: MREC-20- 008.
IRAS project number: 288296. Dated: 13.11.2020 Version 1

## Section 1: Respondent details

*Your details will remain confidential within the research team*

Please click the box to indicate you have read the Participant Information Sheet: ☐

1. About you

| | |
|---|---|
| Respondent name | |
| Your job title | |
| Your organisation (CCG) *Optional* | |
| Telephone contact no. *Optional* | |
| Email address *Optional* | |

## Section 2: EPaCCS in your CCG

1. Is the specific EPaCCS you use limited to your own CCG geography?

   Yes: ☐ No: ☐

   a. If **No**, Please list the other CCGs covered

2. What type of organisation hosts the EPaCCS being used in your CCG?

| | |
|---|---|
| Ambulance service | ☐ |
| CCG Commissioning support unit (CSU) | ☐ |
| GP Community services provider | ☐ |
| Hospice | ☐ |
| Informatics service | ☐ |
| NHS Trust | ☐ |
| Out of hours service | ☐ |
| Urgent care services | ☐ |
| Unknown | ☐ |
| Other please specify | |

## **Section 2: EPaCCS in your CCG** (Continued)

3. What year did the EPaCCS go live? (*if known*)

4. Which clinical software system/s are being used?

| | LEAD clinical software system/s | OTHER system/s that information is shared with |
|---|---|---|
| Adastra | ☐ | ☐ |
| Black Pear | ☐ | ☐ |
| Cerner | ☐ | ☐ |
| Cleo | ☐ | ☐ |
| Co-ordinate My Care | ☐ | ☐ |
| EMIS | ☐ | ☐ |
| Graphnet | ☐ | ☐ |
| Health Analytics | ☐ | ☐ |
| MIG | ☐ | ☐ |
| SCR | ☐ | ☐ |
| SystemC | ☐ | ☐ |
| SystmOne | ☐ | ☐ |
| Vision | ☐ | ☐ |
| To be confirmed | ☐ | ☐ |
| Don't know | ☐ | ☐ |
| Other please specify | | |

5. Was this the first attempt at implementation?

   Yes: ☐ No: ☐ Don’t know: ☐

   a. If **No** - what hindered previous attempts at implementation?

## Section 2: EPaCCS in your CCG (Continued)

6. What is the intended impact for patients of using EPaCCS?

| |
|---|
| |

7. How do you measure the impact for patients?

| |
|---|
| |

## Section 3: Live EPaCCS in your CCG

The questions on the following page relate to data on patients with an EPaCCS record in the area covered by your current EPaCCS solution.

**If you submit this data (where available) - we will provide you with a confidential personalised report of benchmarking data.**

- The data we are requesting is for the period between:
  **1 April 2019 and 31 March 2020**.
- If you are also able to provide data for the period between:
  **1 April 2020 and 30 September 2020** it will enable us to explore how COVID-19 has impacted upon EPaCCS use.

*Please note: for the CCGs in the London area served by Co-ordinate my Care (CMC), a representative has confirmed they are able to obtain this data for each CCG. If you use CMC - please select 1st option below.*

1. **Please indicate if you are unable to provide this activity data yourself and then go to section 4.**

☐ I will nominate a representative (usually a data manager) to provide this
Please enter the name (or state if in London and covered by CMC) and email address of your representative (*their details will remain confidential within the research team*). We will approach them for patient activity data.

| |
|---|
| |

☐ Our systems are not fully operational
☐ We do not have this data

## Section 3: EPaCCS activity data

**How many people**:

| | Number of records 01.04.19 - 31.03.20 | Number of records 01.04.20 - 30.09.20 |
|---|---|---|
| Died? | | |
| Had an EPaCCS record? | | |
| With an EPaCCS record died? | | |
| With an EPaCCS record had anticipatory medications recorded? | | |

**Of those who died with an EPaCCS record, how many died at:**

| | Number of records 01.04.19 - 31.03.20 | Number of records 01.04.20 - 30.09.20 |
|---|---|---|
| Had their preferred place of death recorded? | | |
| Died in their preferred place of death? | | |

**Of those with an EPaCCS record, how many died at:**

| | Number of records 01.04.19 - 31.03.20 | Number of records 01.04.20 - 30.09.20 |
|---|---|---|
| Hospital | | |
| Care Home | | |
| Hospice | | |
| Home | | |
| Other (state) | | |

**Of those who died with an EPaCCS record, how many had:**

| | Number of records 01.04.19 - 31.03.20 | Number of records 01.04.20 - 30.09.20 |
|---|---|---|
| | | |
| A primary diagnosis of cancer? | | |
| Tested positive for SARS-CoV-2 (COVID-19)? | | |

## Section 4: EPaCCS access

1. To the best of your knowledge - please indicate in the table below which care settings have access to the information in the EPaCCS record and in which formats?

| | Type of access available to EPaCCS records | | | |
|---|---|---|---|---|
| | No access | via letter or fax | via co-ordination centre | via shared web interface or viewer |
| GP | ☐ | ☐ | ☐ | ☐ |
| Out of hours GP | ☐ | ☐ | ☐ | ☐ |
| Nurse practitioners assigned to primary care practices | ☐ | ☐ | ☐ | ☐ |
| Community nursing teams | ☐ | ☐ | ☐ | ☐ |
| Palliative care clinical nurse specialists | ☐ | ☐ | ☐ | ☐ |
| Palliative care doctor (hospice or community based) | ☐ | ☐ | ☐ | ☐ |
| Care home staff | ☐ | ☐ | ☐ | ☐ |
| Community social enterprises | ☐ | ☐ | ☐ | ☐ |
| Social care staff | ☐ | ☐ | ☐ | ☐ |
| Ambulance staff | ☐ | ☐ | ☐ | ☐ |
| Hospital palliative care teams (i.e. doctors, specialist nurses) | ☐ | ☐ | ☐ | ☐ |
| Other hospital services (i.e. emergency dept, other hospital teams) | ☐ | ☐ | ☐ | ☐ |
| NHS 111 | ☐ | ☐ | ☐ | ☐ |
| Other, Please state below | | | | |

## Section 4: EPaCCS access (continued)

*(Continued) please indicate which care settings have access to the information in the EPaCCS record and in which formats?*

| | **Type of access available to EPaCCS records** | | |
|---|---|---|---|
| | via automated email | Direct electronic system to system transfer | Batch or overnight electronic transfer |
| GP | ☐ | ☐ | ☐ |
| Out of hours GP | ☐ | ☐ | ☐ |
| Nurse practitioners assigned to primary care practices | ☐ | ☐ | ☐ |
| Community nursing teams | ☐ | ☐ | ☐ |
| Palliative care clinical nurse specialists | ☐ | ☐ | ☐ |
| Palliative care doctor (hospice or community based) | ☐ | ☐ | ☐ |
| Care home staff | ☐ | ☐ | ☐ |
| Community social enterprises | ☐ | ☐ | ☐ |
| Social care staff | ☐ | ☐ | ☐ |
| Ambulance staff | ☐ | ☐ | ☐ |
| Hospital palliative care teams (i.e. doctors, specialist nurses) | ☐ | ☐ | ☐ |
| Other hospital services (i.e. emergency dept, other hospital teams) | ☐ | ☐ | ☐ |
| NHS 111 | ☐ | ☐ | ☐ |
| Other, Please state below | | | |

a. Other - please specify setting/s or health professional group/s and access type/s (if known)

b. Please specify the CCGs that the information in the table relates to

## Section 4: EPaCCS access

1. Do patients have access to their own EPaCCS record?

   Yes: ☐ No: ☐

   a. If **Yes**, What year was patient access enabled?

   b. How is access enabled?
      - ☐ NHS login
      - ☐ Via Android or iOS App
      - ☐ Medical record GP practice
      - ☐ Other, please state

   c. What level of access do patients have?
      - ☐ Read only
      - ☐ Editable
      - ☐ Other, please state

   d. Which items can patients edit?

## Section 4: EPaCCS access

1. What is the annual commissioned spend on EPaCCS, if known, and what does this cover?

| |
|---|
| |

| | Yes | No | If **Yes**, please brief describe |
|---|---|---|---|
| 2. Are there any known additional costs associated with EPaCCS (e.g. monitoring, training, software)? | ☐ | ☐ | |
| 3. Are there (previous, current or planned) processes for monitoring uptake and healthcare professional interaction with EPaCCS across the CCG? | ☐ | ☐ | |
| 4. Have you observed any unforeseen consequences of EPaCCS since implementation? | ☐ | ☐ | |

## Section 5: Barriers & facilitators to EPaCCS development & implementation

1. What clinical challenges have been experienced in the use or implementation of EPaCCS?

| | |
|---|---|
| ☐ | Hard to engage stakeholders (GPs) |
| ☐ | Hard to engage stakeholders (other) |
| ☐ | Administration rights (who can add/administer/ has responsibility) |
| ☐ | Patient consent |
| ☐ | Clinical leadership |
| ☐ | IT leadership |
| ☐ | IT support |
| ☐ | Training support |
| ☐ | Other, please specify: |

2. Please outline any previous, current or planned activities that target clinical staff use and uptake of EPaCCS (e.g. training, personalised engagement with services?

| |
|---|
| |

3. Do you have any needs in your role to support the implementation, monitoring or development of EPaCCS?

   Yes: ☐ No: ☐

   a. If **Yes**, What are your needs and what could be provided to support you in meeting these needs?

| |
|---|
| |

## Section 6: Looking ahead

The national information standard ISB1580 currently defines core content held in EPaCCS or other end of life care co-ordination systems and specifies requirements for its implementation. The standard is due to be revised and information you provide will contribute to its revision.

1. Have you experienced any issues with implementing standard ISB1580?

   Yes: ☐ No: ☐

   a. If **Yes**, what issues have you experienced? e.g. *difficulty implementing the existing standards, development of an EPaCCS in line with SNOMED codes, limited fields available for some items*

2. Have you implemented any other information standards for end of life care services, including any that are locally developed?

   Yes: ☐ No: ☐ Don't know: ☐

3. If **Yes**, would you be happy to share these with us?

| | |
|---|---|
| ☐ | Yes - *Please check you have entered your contact details and we'll send you a request* |
| ☐ | No |

**The revised standard aims to:**

- Reflect clear definitions of what is and is not an EPaCCS
- Clarify data ownership, management and control
- Place the person receiving care and support at the centre of the standards intentions
- Ensure inter-operability and equity of access for all direct care providers and the person receiving care and support
- Require systems to be accessible in a timely manner appropriate to role, circumstance and environment
- Clarify terminology and coding standards
- Determine minimum data set requirements

4. What do you think would be useful to include in the revised standard to achieve the above aims?

## Section 6: Looking ahead (continued)

5. Have you implemented a ReSPECT (Recommended Summary Plan for Emergency Care and Treatment) form in your CCG?

   Yes: ☐ No: ☐ Partial (some settings): ☐

   a. If **Yes**, Have you adapted the ReSPECT form to an electronic version within your patient record system?

      Yes: ☐ No: ☐ Don't know: ☐

      If **Yes**, What is the most recent version of the ReSPECT form have you adapted?

      Version 1: ☐ Version 2: ☐ Version 3: ☐

   b. If known, how does your ReSPECT form currently align with your EPaCCS record?

   c. Please describe any issues or challenges you have experienced when implementing the ReSPECT alongside an operational EPaCCS

6. Apart from ReSPECT and EPaCCS, do you use any other end of life forms in your CCG?

   Yes: ☐ No: ☐ Don't know: ☐

   a. Which form/s do you use?

   b. Have you adapted the form/s to an electronic version within your patient record system?

      Yes: ☐ No: ☐ Don't know: ☐

## Section 6: Looking ahead (continued)

7. Has COVID-19 had an impact on use of EPaCCS?
   Yes: ☐ No: ☐ Don't know: ☐

   If Yes, How has COVID-19 affected the use of EPaCCS?

## Section 7: Feedback

Your feedback is important and we invite you to give any comments you may have about the implementation of an EPaCCS; examples of things that are going well, impact for staff, for people and their families and examples of any difficulties or barriers to implementation encountered. We are also interested in hearing about your future plans or developments.

**And finally:** We want to invite key stakeholder groups to **a future workshop** to build consensus about how EPaCCS should be used nationally and where there are opportunities to improve how they are used. This will take place towards the end of the project in Autumn/Winter 2022-3.

If you are interested in being contacted about this workshop please indicate here: ☐ and ensure you have entered your email address on page 2.

**Thank you for completing this survey**

**Please email it to: Study Researcher**

# CCGs at the planning stage of implementing an EPaCCS

## Electronic Palliative Care Co-ordination Systems (EPaCCS):
### Evaluating their implementation & optimising future service provision

This survey aims to capture progress towards implementing EPaCCS across England. It will help to understand how they are being used and identify how local implementation can be best supported. It will also support future development of the national information standard and national guidance.

This research is being undertaken by researchers at the University of Leeds, Kings College London and the Hull York Medical School. We are working in partnership with NHS England and Improvement and Public Health England.

The survey takes up to **10 minutes** to complete and should be completed by a CCG end of life care lead or a nominated representative.

If you have any questions, please contact:

Chief Investigator

Research Fellow

**Your participation will enable us to provide you with a report of benchmarking data describing models of implementation and reported levels of uptake, and comparing local data with anonymised data from other CCGs that complete the survey.**

This study has been approved by University of Leeds School of Medicine Ethics committee: MREC-20- 008.
IRAS project number: 288296. Dated: 13.11.2020 Version 1

## Section 1: Respondent details

*Your details will remain confidential within the research tea*

Please click the box to indicate you have read the Participant Information Sheet: ☐

2. About you

| | |
|---|---|
| Respondent name | |
| Your job title | |
| Your organisation (CCG) *Optional* | |
| Telephone contact no. *Optional* | |
| Email address *Optional* | |

## Section 2: EPaCCS in your CCG

8. Will your EPaCCS be limited to your own CCG geography?

Yes: ☐ No: ☐

b. If **No**, Please list the other CCGs it will cover

| |
|---|
| |

9. What type of organisation will host the planned EPaCCS in your CCG?

| | |
|---|---|
| Ambulance service | ☐ |
| CCG Commissioning support unit (CSU) | ☐ |
| GP Community services provider | ☐ |
| Hospice | ☐ |
| Informatics service | ☐ |
| NHS Trust | ☐ |
| Out of hours service | ☐ |
| Urgent care services | ☐ |
| Unknown | ☐ |
| Other please specify | |

## **Section 2: EPaCCS in your CCG** (Continued)

10. What (approximately) is the anticipated start date? (*if known*)

| |
|---|
| |

11. Which clinical software system/s will be used?

| | LEAD clinical software system/s | OTHER system/s that information is shared with |
|---|---|---|
| Adastra | ☐ | ☐ |
| Black Pear | ☐ | ☐ |
| Cerner | ☐ | ☐ |
| Cleo | ☐ | ☐ |
| Co-ordinate My Care | ☐ | ☐ |
| EMIS | ☐ | ☐ |
| Graphnet | ☐ | ☐ |
| Health Analytics | ☐ | ☐ |
| MIG | ☐ | ☐ |
| SCR | ☐ | ☐ |
| SystemC | ☐ | ☐ |
| SystmOne | ☐ | ☐ |
| Vision | ☐ | ☐ |
| To be confirmed | ☐ | ☐ |
| Don't know | ☐ | ☐ |
| Other please specify | | |

12. Will this be first attempt at implementation?

Yes: ☐ No: ☐ Don’t know: ☐

b. If **No** - what hindered previous attempts at implementation?

| |
|---|
| |

## Section 2: EPaCCS in your CCG (Continued)

13. What is the intended impact for patients of using EPaCCS?

| |
|---|
| |

14. How will you measure the impact for patients?

| |
|---|
| |

*Section 3 & 4 is for CCGs with an EPaCCS in place and this version of the survey bypasses these parts.*

## Section 5: Barriers & facilitators to EPaCCS development & implementation

4. What clinical challenges have been experienced in the planning and/or implementation of EPaCCS?

| | |
|---|---|
| Hard to engage stakeholders (GPs) | ☐ |
| Hard to engage stakeholders (other) | ☐ |
| Administration rights (who can add/administer/ has responsibility) | ☐ |
| Patient consent | ☐ |
| Clinical leadership | ☐ |
| IT leadership | ☐ |
| IT support | ☐ |
| Training support | ☐ |
| Other, please specify | |

5. Please outline any planned activities that target clinical staff use and uptake of EPaCCS (e.g. training, personalised engagement with services?

| |
|---|
| |

6. Do you have any needs in your role to support the implementation, monitoring or development of EPaCCS?

   Yes: ☐ No: ☐

   e. If **Yes**, What are your needs and what could be provided to support you in meeting these needs?

| |
|---|
| |

## Section 6: Looking ahead

The national information standard ISB1580 currently defines core content held in EPaCCS or other end of life care co-ordination systems and specifies requirements for its implementation. The standard is due to be revised and information you provide will contribute to its revision.

8. Have you experienced any issues with implementing standard ISB1580?

   Yes: ☐ No: ☐

   *b.* If **Yes**, what issues have you experienced? e.g. *difficulty implementing the existing standards, development of an EPaCCS in line with SNOMED codes, limited fields available for some items*

9. Are you planning to implement any other information standards for end of life care services, including any that are locally developed?

Yes: ☐ No: ☐ Don't know: ☐

10. If **Yes**, would you be happy to share these with us?

| | |
|---|---|
| ☐ | Yes - *Please check you have entered your contact details and we'll send you a request* |
| ☐ | No |

**The revised standard aims to:**

- Reflect clear definitions of what is and is not an EPaCCS
- Clarify data ownership, management and control
- Place the person receiving care and support at the centre of the standards intentions
- Ensure inter-operability and equity of access for all direct care providers and the person receiving care and support
- Require systems to be accessible in a timely manner appropriate to role, circumstance and environment
- Clarify terminology and coding standards
- Determine minimum data set requirements

11. What do you think would be useful to include in the revised standard to achieve the above aims?

## Section 6: Looking ahead (continued)

12. Do you have a ReSPECT (Recommended Summary Plan for Emergency Care and Treatment) form in use in your CCG?

    Yes: ☐ No: ☐ Partial (some settings): ☐

    d. If **YES**, Are you planning to adapt the ReSPECT form to an electronic version within your patient record system?

       Yes: ☐ No: ☐ Don't know: ☐

13. If known, how will your ReSPECT form align with your EPaCCS record?

| |
|---|
| |

Apart from ReSPECT and EPaCCS, do you use any other end of life forms in your CCG?

Yes: ☐ No: ☐ Don't know: ☐

c. Which form/s do you use?

| |
|---|
| |

d. Will you adapt the form/s to an electronic version within your patient record system?

Yes: ☐ No: ☐ Don't know: ☐

## Section 7: Feedback

Your feedback is important and we invite you to give any comments you may have about the implementation of an EPaCCS; examples of things that are going well, impact for staff, for people and their families and examples of any difficulties or barriers to implementation encountered. We are also interested in hearing about your future plans or developments.

| |
|---|
| |

**And finally:** We want to invite key stakeholder groups to **a future workshop** to build consensus about how EPaCCS should be used nationally and where there are opportunities to improve how they are used. This will take place towards the end of the project in Autumn/Winter 2022-3.

If you are interested in being contacted about this workshop please indicate here: ☐ and ensure you have entered your email address on page 2.

**Thank you for completing this survey**

**Please email it to: Study Researcher**

# CCGs without an EPaCCS

## Electronic Palliative Care Co-ordination Systems (EPaCCS):
### Evaluating their implementation & optimising future service provision

This survey aims to capture progress towards implementing EPaCCS across England. It will help to understand how they are being used and identify how local implementation can be best supported. It will also support future development of the national information standard and national guidance.

This research is being undertaken by researchers at the University of Leeds, Kings College London and the Hull York Medical School. We are working in partnership with NHS England and Improvement and Public Health England.

**The survey takes less than 5 minutes and should be completed by a CCG end of life care lead or a nominated representative.**

If you have any questions, please contact:

Chief Investigator

Research Fellow

This study has been approved by University of Leeds School of Medicine Ethics committee: MREC-20- 008.
IRAS project number: 288296. Dated: 13.11.2020 Version 1

## Section 1: Respondent details

*Your details will remain confidential within the research team*

**Please click the box to indicate you have read the Participant Information Sheet:** ☐

| | |
|---|---|
| Respondent name | |
| Your job title | |
| Your organisation (CCG) *Optional* | |
| Telephone contact number | |
| Email address *Optional* | |

Have you previously attempted implementation?

Yes**:** ☐ No**:** ☐ Don't know**:** ☐

If **Yes** - what hindered previous attempts at implementation?

Your feedback is important and we invite you to give any other comments you may have about the implementation of an EPaCCS

**And finally:** We want to invite key stakeholder groups to **a future workshop** to build consensus about how EPaCCS should be used nationally and where there are opportunities to improve how they are used. This will take place towards the end of the project in Autumn/Winter 2022-3.

If you are interested in being contacted about this workshop please indicate here:

☐ and ensure you have entered your email address above.

**Thank you for completing this survey - please email it to: Study Researcher**
