# Supplementary material for: Mapping and characterising electronic palliative care coordination systems and their intended impact: A national survey of end-of-life care commissioners
Source: PLoS One. 2022 Oct 14;17(10):e0275991. doi: 10.1371/journal.pone.0275991 (PMC9565729; doi:10.1371/journal.pone.0275991)
Supplement: S1 Table — (PDF) [file pone.0275991.s003.pdf]

Electronic patient record system providers involved in storage and sharing of data captured in EPaCCS

System	CCGs with Operational EPaCCS		CCGs planning an EPaCCS	
	Lead system	Other system	Lead system	Other system
SystmOne	28	16	3	5
EMIS	27	17	4	7
Co-ordinate My Care	18		3	
Black Pear	7	2		1
Cerner	6	3		
Adastra	5	21	2	6
MIG	3	8	2	1
Graphnet	2	2	5	
Vision	1	1		3
Bespoke local system		11		4
SCR		8		3
GP Connect		2		
icare		2		
Care IS		1		
Rio		1		1
Care and Health Information Exchange (CHIE)		1		
e-healthscope		1		
Lorenzo		1	2	
Cleric				2
Don't know			2	
