# Supplementary material for: Mapping and characterising electronic palliative care coordination systems and their intended impact: A national survey of end-of-life care commissioners
Source: PLoS One. 2022 Oct 14;17(10):e0275991. doi: 10.1371/journal.pone.0275991 (PMC9565729; doi:10.1371/journal.pone.0275991)
Supplement: S2 Table — (PDF) [file pone.0275991.s004.pdf]

Care settings and levels of access to EPaCCS as reported by CCGs with an operational system

Setting	Service (*n* of responses received for setting)	Shared web interface or viewer (%)	Direct electronic system to system transfer (%)	No access (%)
Community settings	General practitioners (n=56)	55*	52*	0
	Out of hours GP (n=53)	60*	36*	0
	Practice-based nurses (n=51)	57*	43*	4
	Community nursing teams (n=51)	55*	47*	6
	Community-based palliative care teams (n=55)	64	42	2
	Care homes (n=44)	16	5	61
	Community social enterprises (n=40)	8	8	85
	Social care staff (n=42)	17	7	67
	Ambulance staff (n=46)	52	7	17
Hospital settings	Hospital palliative care teams (n=52)	67*	35*	2
	Other hospital services (n=46)	57	33	7
Urgent care	NHS 111 (n=46)	48	17	22

Key: *Percentage (%) reported access is derived from number of CCGs reporting access as a proportion of the number of CCGs providing a response. Of 57 respondents, 56 CCGs indicate a method of access for any of the services, with 23 CCGs providing data for some and not all health professional groups. In some CCGs, more than one method of sharing information was selected for the same service type.*Respondents that selected both "Shared web interface or viewer" & "Direct electronic system to system transfer" are represented in each of these columns. These were General practitioners (4/56), Out of hours GP (3/53), Nurse practitioners (3/51), Community nursing teams (5/51), Hospital palliative care teams (4/52). "Shared web interface" is defined as software that is used over the internet with a web browser, where data is typically stored on the cloud. This approach also includes standalone web-based platforms that gather and present data from a range of existing electronic patient record systems within a defined geographical boundary. "Direct electronic system to system transfer" refers to server-based software that is installed on often remote servers, with access to an EPaCCS record achieved through a patient's existing electronic patient record system (e.g. in a primary care practice) and edits made to the record are synchronised with other, related electronic patient record systems. Other types of access to EPaCCS were reported – these included: batch or overnight electronic transfer, access via a coordination centre, information shared by automated email, and information sent by letter or fax. These methods were more likely to be used by ambulance staff (11 of 46 responses), care homes (8 of 44 responses) and NHS 111 (6 of 46 responses) which is a national telephone and web service supporting urgent and emergency care.*
