# Supplementary material for: Mapping and characterising electronic palliative care coordination systems and their intended impact: A national survey of end-of-life care commissioners
Source: PLoS One. 2022 Oct 14;17(10):e0275991. doi: 10.1371/journal.pone.0275991 (PMC9565729; doi:10.1371/journal.pone.0275991)
Supplement: S3 Table — (PDF) [file pone.0275991.s005.pdf]

Alignment of intended impact with measurement reported by survey respondents

CCGs with operational EPaCCS

CCG No.	Intended Impact	Alignment with measure
2	Care coordination	Not measured
3	Care coordination	Not measured
5	Care coordination	Low
	Patient impact - Meet wishes	Low
	Patient impact - Better conversations	Low
6	Care coordination	High
	Patient impact - Meet wishes	High
7	Care coordination	High
	Patient impact - Meet wishes	High
14	Care coordination	Low
	Patient impact - Meet wishes	Low
15	Access to information	Low
20	Care coordination	Low
	Access to information	Low
22	Patient impact - Meet wishes	Low
23	Patient impact - Meet wishes	Low
24	Patient impact - Meet wishes	Low
25	Patient impact - Meet wishes	Low
26	Patient impact - Meet wishes	Low
27	Care coordination	Low
28	Access to information	High
30	Access to information	Not measured
	Care coordination	Not measured
	Patient impact - Better conversations	Not measured
	Patient impact - Meet wishes	Not measured
	HP Practice	Not measured
32	Patient impact - Better conversations	Low
37	Patient impact - Better conversations	Low
39	Patient impact - Meet wishes	High
40	Patient impact - Better conversations	High
	Access to information	Low
43	Care coordination	Not measured
44	Care coordination	Not measured
46	Patient impact - Better conversations	Low
	Patient impact - Meet wishes	Low
47	Patient impact - Better conversations	Low
	Patient impact - Meet wishes	Low
48	Access to information	Low
	Patient impact - Meet wishes	Low
50	Access to information	High
	Patient impact - Better conversations	High
	Patient impact - Meet wishes	High
51	Access to information	High
	Patient impact - Better conversations	High
	Patient impact - Meet wishes	High
52	Access to information	High
	Patient impact - Better conversations	High

CCG No.	Intended Impact	Alignment with measure
	Patient impact - Meet wishes	High
53	Access to information	High
	Patient impact - Better conversations	High
	Patient impact - Meet wishes	High
54	Access to information	Low
55	Access to information	Not measured
57	Care coordination	Low
	Access to information	Low
	Patient impact - Meet wishes	Low
58	HP Practice	Low
	Patient impact - Better conversations	Low
	Care coordination	Low
	Patient impact - Meet wishes	Low
59	HP Practice	Low
	Patient impact - Better conversations	Low
	Care coordination	Low
	Patient impact - Meet wishes	Low
61	Patient impact - Meet wishes	Low
62	Care coordination	High
	Access to information	High
	Patient impact - Meet wishes	High
	Patient impact - Better conversations	High
	Family impacts	High
63	Care coordination	High
	Access to information	High
	Patient impact - Meet wishes	High
	Patient impact - Better conversations	High
	Family impacts	High
64	Care coordination	High
	Access to information	High
	Patient impact - Meet wishes	High
	Patient impact - Better conversations	High
	Family impacts	High
65	Care coordination	High
	Access to information	High
	Patient impact - Meet wishes	High
	Patient impact - Better conversations	High
	Family impacts	High
66	Care coordination	High
	Access to information	High
	Patient impact - Meet wishes	High
	Family impacts	High
	Patient impact - Better conversations	High
67	Care coordination	High
	Access to information	High
	Patient impact - Meet wishes	High
	Patient impact - Better conversations	High
	Family impacts	High
68	Care coordination	High
	Access to information	High

CCG No.	Intended Impact	Alignment with measure
	Patient impact - Meet wishes	High
	Patient impact - Better conversations	High
	Family impacts	High
69	Care coordination	High
	Access to information	High
	Patient impact - Meet wishes	High
	Patient impact - Better conversations	High
	Family impacts	High
70	Access to information	Low
	Patient impact - Meet wishes	Low
71	Care coordination	Low
	Patient impact - Meet wishes	Low
	Access to information	Low
72	Access to information	High
73	Access to information	Low
	Patient impact - Meet wishes	Low
	Family impacts	Low
75	Family impacts	Low
	Patient impact - Meet wishes	Low
76	Patient impact - Meet wishes	Low
	Access to information	Low
	Patient impact - Better conversations	Low
77	Care coordination	High
	Patient impact - Meet wishes	Low
79	Access to information	Low
80	Care coordination	Not measured
81	Access to information	High
	Patient impact - Meet wishes	Low
82	Care coordination	High
	Patient impact - Meet wishes	Low
	Family impacts	Low
83	Care coordination	High
	Patient impact - Meet wishes	Low
	Family impacts	Low
84	Care coordination	Low
	Patient impact - Meet wishes	Low
85	HP Practice	Not measured
	Care coordination	Not measured

CCGs Planning an EPaCCS

CCG No.	Intended Impact	Alignment with measure
1	Access to information	Not measured
10	Patient impact - Meet wishes	High
	Care coordination	High
11	Patient impact - Better conversations	High
	Patient impact - Meet wishes	High
12	Care coordination	Low
13	Care coordination	Low

CCG No.	Intended Impact	Alignment with measure
19	Patient impact - Meet wishes	High
	Care coordination	Low
	Patient impact - Better conversations	High
	Access to information	Low
35	Care coordination	Low
	Patient impact - Meet wishes	Low
36	Care coordination	Low
	Patient impact - Meet wishes	Low
	Access to information	Low
38	Patient impact - Meet wishes	High
	Care coordination	High
41	Family impacts	Low
	Patient impact - Meet wishes	Low
42	Care coordination	Low
	Patient impact - Meet wishes	Low
49	Care coordination	Low
	Patient impact - Meet wishes	Low
60	No impact stated	not applicable
