# Supplementary material for: Mapping and characterising electronic palliative care coordination systems and their intended impact: A national survey of end-of-life care commissioners
Source: PLoS One. 2022 Oct 14;17(10):e0275991. doi: 10.1371/journal.pone.0275991 (PMC9565729; doi:10.1371/journal.pone.0275991)
Supplement: Attachment — Submitted filename: Response to reviewers.docx [file pone.0275991.s006.docx]

We would like to thank the reviewers and editorial team for their time and effort when appraising our manuscript. Please find below a copy of our response to points raised by the reviewers alongside details of how we have addressed them in the manuscript.

| **Reviewer comment** | **Author response** |
| --- | --- |
| **Reviewer 1** | |
| Thank you for the opportunity to review this manuscript that examines an important policy translation issue in palliative care in UK. The paper is a well thought-out and well-written one. I have very minor comments. | Many thanks for your time and kind and helpful comments when undertaking the review. |
| Results highlighted that 82 individuals responded on behalf of 85 CCGs. Does this infer that some participants reported on behalf of more than one CCG? I think this needs to be clearly stated and the implications explored. | We have included the following text to clarify this point in the findings section: “Responses for multiple CCGs by one respondent occurred in areas where delivery of palliative and end of life care is overseen across an Integrated Care System (a statutory partnership of organisations providing care across a geographical area that encompass more than one CCG).” In terms of the implications, palliative and end of life care leads within an Integrated Care System would be able to describe the approach adopted to host an Electronic Palliative Care Coordination System across the CCGs comprising an Integrated Care System. From the responses we received, the EPaCCS approach within each Integrated Care System was harmonised across all CCGs contained within them so that one respondent could consistently report the EPaCCS approach for multiple CCGs. |
| Line 13 on Page 7 reads: 'For operational CCGs, nine different electronic patient record software products were reported as used to host EPaCCS.' This sentence needs to be rewritten for clarity. | Thank you for pointing out where the text was unclear. We have rewritten the sentence to read: “Across CCGs with operational EPaCCS, nine different electronic health record products were identified as being used to support the capture, storage or sharing of data.” |
| **Reviewer 2** | |
| Thank you for giving me opportunity for reviewing this paper. It is an important article about use and impact of electronic palliative care co-ordination system. However, I have some recommendation regarding the manuscript. | Thank you for your comments and helpful suggestions on how we can improve the manuscript. |
| 1. In the abstract, it would be better if you mention from whom or where you have collected the data (eg. health care providers/ patients etc) in the methods section.  2.Same comment goes for the recruitment and sample section of the methodology |  |
|  | Thank you for your comment. We have clarified who we collected data from in the methods section with the following text:  “We surveyed all commissioning leads in palliative and end-of-life care in each Clinical Commissioning Group (CCG) in England (n=135). Commissioning leads are typically either or both: i) a clinical lead, typically a GP, or; ii) a managerial lead in terms of the lead commissioning manager.”  And we have ensured that this is referred to in the methods section of the abstract:  “We conducted a national cross-sectional online survey of end-of-life care commissioning leads for Clinical Commissioning Groups (CCGs) in England”. |
| 3. Have you conducted any statistical testes in the tables no 1 and 2. Some statistical tests can make your results more stronger | Thank you for your suggestion. This work was undertaken with the purpose of determining the current implementation status of EPaCCS, their role in information sharing and intended impact, and requested routine patient-level data relating to EPaCCS. This lends itself to descriptive analysis which we had planned in our study protocol and present in the manuscript. We used descriptive statistics using SPSS to outline levels of uptake, descriptive comparisons made between models of implementation and their intended impact, and used geographically mapping software to undertake analysis of geographical differences in stages and models of EPaCCS implementation. The use of statistical tests, such as factors that may influence how many people have an EPaCCS record prior to death are being developed by our group in planned follow-on research. |
| 4. I think table 3 in the discussion section can be moved to the appendix section. Also the points inside the tables can be described under the heading of strength and limitations | Thank you for this suggestion. We included Table 3 in the discussion to synthesise the context prior to this study, key study findings, and their implications for research, practice and policy, to assist the reader in summarising the research. We feel that this would be missed by readers as an appendix and would be better to remain in the discussion section, but can refer to the Editor for a decision on its placement. |
| **Journal requirements** | |
| 1. Please ensure that your manuscript meets PLOS ONE's style requirements, including those for file naming. | The formatting and labelling of documents have been amended to meet the style requirements. |
| 2. Please provide additional details regarding participant consent. In the ethics statement in the Methods and online submission information, please ensure that you have specified what type you obtained (for instance, written or verbal, and if verbal, how it was documented and witnessed). If your study included minors, state whether you obtained consent from parents or guardians. If the need for consent was waived by the ethics committee, please include this information. | We have added the following text to the Methods section (page 5) to ensure this is clear: “Participants were first presented with information about the study and its aims and a consent form. Participants were required to provide written consent to participate prior to accessing the survey content.” We have also specified that written consent was provided in the Ethics statement on the manuscript submission system and in the Declarations document. |
| 3. Thank you for stating the following financial disclosure:  This study is funded by the National Institute for Health Research (NIHR) [Health Services and Delivery Research (NIHR129171)]. The views expressed are those of the authors and not necessarily those of the NIHR or the Department of Health and Social Care.    Please state what role the funders took in the study. If the funders had no role, please state: "The funders had no role in study design, data collection and analysis, decision to publish, or preparation of the manuscript."  If this statement is not correct you must amend it as needed.  Please include this amended Role of Funder statement in your cover letter; we will change the online submission form on your behalf. | We have included the following in the funding statement in the Declarations document: "The funders had no role in study design, data collection and analysis, decision to publish, or preparation of the manuscript." |
| 4. We note that you have indicated that data from this study are available upon request. PLOS only allows data to be available upon request if there are legal or ethical restrictions on sharing data publicly. For more information on unacceptable data access restrictions, please see <http://journals.plos.org/plosone/s/data-availability#loc-unacceptable-data-access-restrictions>.  In your revised cover letter, please address the following prompts:  a) If there are ethical or legal restrictions on sharing a de-identified data set, please explain them in detail (e.g., data contain potentially sensitive information, data are owned by a third-party organization, etc.) and who has imposed them (e.g., an ethics committee). Please also provide contact information for a data access committee, ethics committee, or other institutional body to which data requests may be sent.  b) If there are no restrictions, please upload the minimal anonymized data set necessary to replicate your study findings as either Supporting Information files or to a stable, public repository and provide us with the relevant URLs, DOIs, or accession numbers. For a list of acceptable repositories, please see <http://journals.plos.org/plosone/s/data-availability#loc-recommended-repositories>.  We will update your Data Availability statement on your behalf to reflect the information you provide. | We have included the following detail in our revised cover letter:  “Pseudonymized data are stored on a secure server at the University of Leeds. Access to aggregated data can be provided by the corresponding author upon request after applying measures to minimize risk of reidentification and approval from the responsible data protection officer. Due to the sensitive information contained, restrictions to data have been mandated by the National Health Service Health Research Authority and Health and Care Research Wales who oversaw ethical approval of our research. Requests for a de-identified dataset underpinning the findings presented in the manuscript can be made to the corresponding author via email ([m.j.allsop@leeds.ac.uk](mailto:m.j.allsop@leeds.ac.uk)) which will be sent to the National Health Service Health Research Authority and Health and Care Research Wales for review.” |
| 5. Please include your full ethics statement in the ‘Methods’ section of your manuscript file. In your statement, please include the full name of the IRB or ethics committee who approved or waived your study, as well as whether or not you obtained informed written or verbal consent. If consent was waived for your study, please include this information in your statement as well. | We have included the full ethics statement in the Methods sections including the full name of the ethics committee and specified that written consent was provided. |
| 6. We note that Figure 1 in your submission contain [map/satellite] images which may be copyrighted. All PLOS content is published under the Creative Commons Attribution License (CC BY 4.0), which means that the manuscript, images, and Supporting Information files will be freely available online, and any third party is permitted to access, download, copy, distribute, and use these materials in any way, even commercially, with proper attribution. For these reasons, we cannot publish previously copyrighted maps or satellite images created using proprietary data, such as Google software (Google Maps, Street View, and Earth). For more information, see our copyright guidelines: <http://journals.plos.org/plosone/s/licenses-and-copyright>.   We require you to either (1) present written permission from the copyright holder to publish these figures specifically under the CC BY 4.0 license, or (2) remove the figures from your submission:   a. You may seek permission from the original copyright holder of Figure 1 to publish the content specifically under the CC BY 4.0 license.     We recommend that you contact the original copyright holder with the Content Permission Form (<http://journals.plos.org/plosone/s/file?id=7c09/content-permission-form.pdf>) and the following text: “I request permission for the open-access journal PLOS ONE to publish XXX under the Creative Commons Attribution License (CCAL) CC BY 4.0 (<http://creativecommons.org/licenses/by/4.0/>). Please be aware that this license allows unrestricted use and distribution, even commercially, by third parties. Please reply and provide explicit written permission to publish XXX under a CC BY license and complete the attached form.”   Please upload the completed Content Permission Form or other proof of granted permissions as an "Other" file with your submission.   In the figure caption of the copyrighted figure, please include the following text: “Reprinted from [ref] under a CC BY license, with permission from [name of publisher], original copyright [original copyright year].”   b. If you are unable to obtain permission from the original copyright holder to publish these figures under the CC BY 4.0 license or if the copyright holder’s requirements are incompatible with the CC BY 4.0 license, please either i) remove the figure or ii) supply a replacement figure that complies with the CC BY 4.0 license. Please check copyright information on all replacement figures and update the figure caption with source information. If applicable, please specify in the figure caption text when a figure is similar but not identical to the original image and is therefore for illustrative purposes only. The following resources for replacing copyrighted map figures may be helpful:   USGS National Map Viewer (public domain): <http://viewer.nationalmap.gov/viewer/> The Gateway to Astronaut Photography of Earth (public domain): <http://eol.jsc.nasa.gov/sseop/clickmap/> Maps at the CIA (public domain): <https://www.cia.gov/library/publications/the-world-factbook/index.html> and <https://www.cia.gov/library/publications/cia-maps-publications/index.html> NASA Earth Observatory (public domain): <http://earthobservatory.nasa.gov/> Landsat: <http://landsat.visibleearth.nasa.gov/> USGS EROS (Earth Resources Observatory and Science (EROS) Center) (public domain): [http://eros.usgs.gov/#](http://eros.usgs.gov/) Natural Earth (public domain): <http://www.naturalearthdata.com/> | The boundaries used in Figure 1 were generated from a feature layer file generated by the UK Office for National Statistics (ONS). if you wish to use or re-use ONS material, whether commercially or privately, you may do so freely without a specific application for a licence, subject to the conditions of the Open Government Licence and the Framework. If you are reproducing ONS content you must include a source accreditation to ONS only: <https://www.ons.gov.uk/methodology/geography/licences>  As aligned with ONS licence expectations, we have included the following details in the Methods section under analysis where the development of Figure 1 is detailed: “The location of respondents and the status of their EPaCCS were plotted on a boundary and reference map (Clinical Commissioning Groups (April 2021) EN BUC) generated by the UK Office for National Statistics (Source: Office for National Statistics licensed under the Open Government Licence v.3.0; Contains OS data © Crown copyright and database right 2022.” |
| 7. Please include captions for your Supporting Information files at the end of your manuscript, and update any in-text citations to match accordingly. Please see our Supporting Information guidelines for more information: <http://journals.plos.org/plosone/s/supporting-information>. | This have been included at the end of the manuscript document. |
| 8. Please review your reference list to ensure that it is complete and correct. If you have cited papers that have been retracted, please include the rationale for doing so in the manuscript text, or remove these references and replace them with relevant current references. Any changes to the reference list should be mentioned in the rebuttal letter that accompanies your revised manuscript. If you need to cite a retracted article, indicate the article’s retracted status in the References list and also include a citation and full reference for the retraction notice. | We have reviewed the reference list to ensure it is complete and correct and do not include any articles that have subsequently been retracted. |
